# Probing the Mutational Interplay between Primary and Promiscuous Protein Functions: A Computational-Experimental Approach

**DOI:** 10.1371/journal.pcbi.1002558

**Published:** 2012-06-14

**Authors:** Hector Garcia-Seisdedos, Beatriz Ibarra-Molero, Jose M. Sanchez-Ruiz

**Affiliations:** Facultad de Ciencias, Departamento de Quimica Fisica, Universidad de Granada, Granada, Spain; Stockholm University, Sweden

## Abstract

Protein promiscuity is of considerable interest due its role in adaptive metabolic plasticity, its fundamental connection with molecular evolution and also because of its biotechnological applications. Current views on the relation between primary and promiscuous protein activities stem largely from laboratory evolution experiments aimed at increasing promiscuous activity levels. Here, on the other hand, we attempt to assess the main features of the simultaneous modulation of the primary and promiscuous functions during the course of natural evolution. The computational/experimental approach we propose for this task involves the following steps: a function-targeted, statistical coupling analysis of evolutionary data is used to determine a set of positions likely linked to the recruitment of a promiscuous activity for a new function; a combinatorial library of mutations on this set of positions is prepared and screened for both, the primary and the promiscuous activities; a partial-least-squares reconstruction of the full combinatorial space is carried out; finally, an approximation to the Pareto set of variants with optimal primary/promiscuous activities is derived. Application of the approach to the emergence of folding catalysis in thioredoxin scaffolds reveals an unanticipated scenario: diverse patterns of primary/promiscuous activity modulation are possible, including a moderate (but likely significant in a biological context) simultaneous enhancement of both activities. We show that this scenario can be most simply explained on the basis of the conformational diversity hypothesis, although alternative interpretations cannot be ruled out. Overall, the results reported may help clarify the mechanisms of the evolution of new functions. From a different viewpoint, the partial-least-squares-reconstruction/Pareto-set-prediction approach we have introduced provides the computational basis for an efficient directed-evolution protocol aimed at the simultaneous enhancement of several protein features and should therefore open new possibilities in the engineering of multi-functional enzymes.

## Author Summary

Interpretations of evolutionary processes at the molecular level have been determined to a significant extent by the concept of “trade-off”, the idea that improving a given feature of a protein molecule by mutation will likely bring about deterioration in other features. For instance, if a protein is able to carry out two different molecular tasks based on the same functional site (competing tasks), optimization for one task could be naively expected to impair its performance for the other task. In this work, we report a computational/experimental approach to assess the potential patterns of modulation of two competing molecular tasks in the course of natural evolution. Contrary to the naïve expectation, we find that diverse modulation patterns are possible, including the simultaneous optimization of the two tasks. We show, however, that this simultaneous optimization is not in conflict with the trade-offs expected for two competing tasks: using the language of the theory of economic efficiency, trade-offs are realized in the Pareto set of optimal variants for the two tasks, while most protein variants do not belong to such Pareto set. That is, most protein variants are not Pareto-efficient and can potentially be improved in terms of several features.

## Introduction

Proteins are capable to perform molecular tasks with impressive efficiency and, often, with exquisite specificity. Nevertheless, many proteins possess weak promiscuous functions, which are more or less related to the primary activity, but involve different substrates or different chemical alterations [1]–[5]. Protein promiscuity has been extensively studied in recent years due to its important biotechnological applications [6]–[12], to its role in adaptive metabolic plasticity [13]–[15] and also because of its fundamental connection with molecular evolution. Indeed, promiscuity in modern proteins is plausibly a vestige of the broad specificity of primordial proteins [1]. Furthermore, as briefly elaborated below, promiscuity likely plays an essential role in the development of new functions through divergent evolution [3], [5], [8], [16]–[20].

Development of new functions does occur during evolution, sometimes with impressive speed. In most cases, the process involves gene duplication as a necessary step. It has been repeatedly noted, however, that random accumulation of mutations in a gene is unlikely to create a new function. It is generally assumed, therefore, that a sufficient level of the new (initially promiscuous) activity must be present before the duplication event. In this way, natural selection can act on one of the gene copies to enhance the new function, while the original function is retained by the other copy. However, optimization of a functional site for a given molecular task likely interferes with the efficient performance of the protein for a different task based on the same site. Consequently, enhancement of the promiscuous activity prior to gene duplication may be expected to cause a decrease in primary activity that could conceivably compromise organism survival. As a solution to this conundrum, a “weak trade-off” scenario has been proposed [5]: enhancement of the promiscuous activity is assumed to be accompanied with only a moderate decrease in primary function and, therefore, a generalist protein (significant levels of both activities) can be formed prior to gene duplication without seriously impairing organism fitness. This weak trade-off explanation is certainly supported by a number of laboratory evolution experiments [5]. However, the possibility that *natural* evolution may actually avoid or bypass primary/promiscuous activity trade-offs (i.e., a “no trade-off” scenario as opposed to a “weak trade-off” scenario) should be seriously taken into account, since bifunctional enzymes with the capability to catalyze efficiently two different biochemical reactions based on the same active site are known and have been recently characterized in detail [21], [22] and experimental studies have supported that the trade-off between high activity and tight specificity can be greatly relaxed [23].

Here, we aim at assessing the patterns of primary/promiscuous activity modulation in the mutational space actually explored by natural evolution when recruiting a promiscuous activity for a new function. The approach we propose involves essentially three steps:

1. *A set of positions that are likely linked with the recruitment of the promiscuous activity for a new function is determined*. For this task we resort to statistical coupling analysis (SCA), a sequence-based bioinformatics procedure originally developed to determine networks of energetically-coupled, co-evolving residues in proteins [24]. SCA has been successfully applied to the design of allosteric communication in proteins [25] and to the design of artificial sequences able to fold to target structures [26].
2. *A combinatorial library of mutations on the set of positions determined in step 1 is prepared and assessed for both, the primary and the promiscuous activities*. In this regard, one methodological (but relevant) point must be made. Since high throughput screening is not generally available, we actually analyze a comparative small number of library variants and use a partial least-squares fitting to these data to reconstruct the whole combinatorial space. Partial least squares (PLS) [27] is a regression technique that allows the prediction of dependent variables (primary and promiscuous activities for the library variants, in the case of interest here) from a very large number of independent variables (related, in the case of interest here, to the individual mutation effects and to non-additivity –i.e., coupling- between mutation effects). PLS has been used in social sciences and chemometrics for many years and its application to protein design has been recently explored [28].
3. *An approximation to the Pareto set of variants of optimal primary/promiscuous activities is obtained from the library screening in order to assess the limits to the simultaneous enhancement of both activities*. The determination of the Pareto set is actually the key goal in the approach we propose. Therefore, we describe below in some detail the meaning of this concept and how it can be used to clarify the relation between the primary and promiscuous activities of a protein.

**Figure 1:**
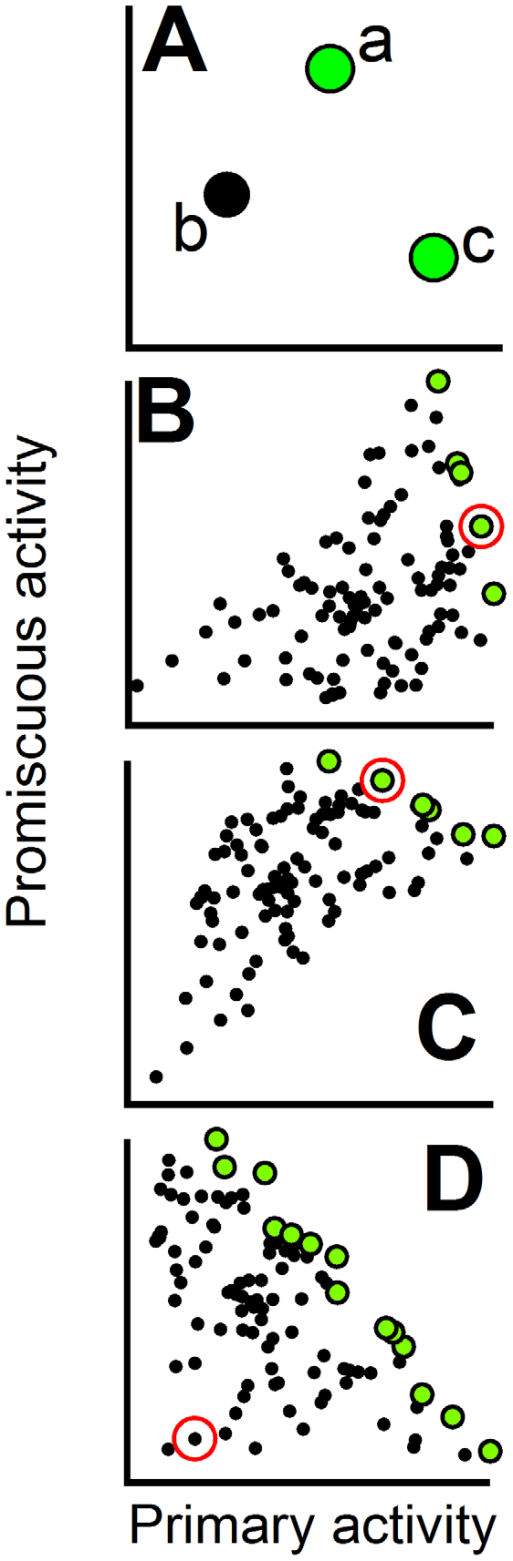
Use of the Pareto set to define the patterns of primary/promiscuous activity modulation. In all plots, each data point represents the primary and promiscuous activity data for a given protein variant. Different variants may be thought as corresponding to different combinations of mutations from a given set. (A) Illustration of Pareto set construction. Variant “b” is dominated by variant “a”, since the latter shows higher values of *both* activities. Variant “a” is not dominated by variant “c”, since promiscuous-activity(a)>promiscuous-activity(c). Variant “c” has the highest value for the primary activity and is not dominated neither by “a” nor “b”. The non-dominated variants “a” and “c” form the Pareto set (green data points) for this three-variant example. (B), (C) and (D) are illustrative examples of the relation between the Pareto set (green data points) and the primary/promiscuous trade-offs. In (B) and (C) the starting variant (marked with a red circle) belongs to the Pareto set and, therefore, increasing the promiscuous activity necessarily implies a decrease of the primary activity. The plot in (B) is meant to illustrate a weak trade-off along the Pareto set (a significant increase in promiscuous activity can be achieved with only a small decrease in primary activity) while (C) is meant to illustrate a strong trade-off. In (D) the starting variant does *not* belong to the Pareto set and, hence, the simultaneous enhancement of both activities is possible.

A unique global optimum cannot be defined when dealing with a multi-objective optimization problem, such as, for instance, enhancing a promiscuous activity while keeping the level of the primary activity as high as possible. However, a set of several optimal solutions can be defined using the Pareto criterion: a solution (protein variant in this case) belongs to the set of optimal solutions (the so-called Pareto set) if it is not *dominated* by any other solution. The dominance relationship is defined as follows: a solution **a** dominates a solution **b** if it shows enhanced performance for all optimization objectives. In the specific case of interest here, variant **a** dominates variant **b** if primary-activity(**a**)>primary-activity(**b**) and *simultaneously* promiscuous-activity(**a**)>promiscuous-activity(**b**). The construction of the Pareto set of non-dominated solutions is illustrated with a simple example in Figure 1A. The Pareto set includes the solutions with optimal trade-offs between the different objectives and has been used extensively in economics, while its application to protein design has only been explored in recent years [29]–[31]. In the specific case of interest here, determination of the Pareto set should immediately clarify the main features of the modulation of the primary and promiscuous activities within a given mutational space. For instance, if the starting variant (the “wild-type” protein, for instance) already belongs to the Pareto set, enhancement of the promiscuous activity necessarily implies a decrease in primary function and the trade-off will be weak (Figure 1B) or strong (Figure 1C) depending of the general slope of the Pareto set in the plot of promiscuous activity versus primary activity. On the other hand, if the starting variant does not belong to the Pareto set, simultaneous optimization of both activities is in principle feasible (Figure 1D) and primary/promiscuous trade-offs can be avoided to some extent.

To test the approach proposed, we have chosen the three basic activities associated with the thioredoxin fold: reduction of disulfide bridges, formation of disulfide bridges and isomerization (reshuffling) of disulfide bridges. The two latter activities are linked in vivo to protein folding processes [32], [33] (oxidative folding and rescuing of proteins with incorrect disulfide bridges) and, in the periplasm of bacteria, are performed by different proteins: DsbA and DsbC, respectively. By contrast, in the endoplasmic reticulum of eukaryotic cells, both disulfide-linked folding processes are catalyzed by the same protein: protein disulfide isomerase (PDI). PDIs are multidomain proteins that contain thioredoxin-fold domains [32]. Processes of disulfide reduction in vivo (obviously unrelated with folding) are typically catalyzed by single-domain thioredoxins, which may also show low in vitro levels of the protein folding activities associated to disulfide bridge formation and reshuffling. Indeed, it is tempting to speculate that low levels of these activities were already present in primordial thioredoxins and that, at some evolutionary point, were recruited for new-function development leading to the proteins involved in disulfide-bridge-linked protein folding [32].

**Figure 2:**
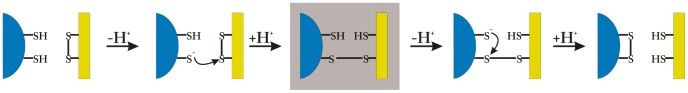
Basic mechanism of disulfide reduction catalyzed by a thioredoxin-fold domain. (Thioredoxin domain shown in blue). The mixed disulfide intermediate has been highlighted in gray. Note the resolution of this intermediate through attack of the thiolate form of the C-terminal cysteine group of the catalyst.

**Figure 3:**
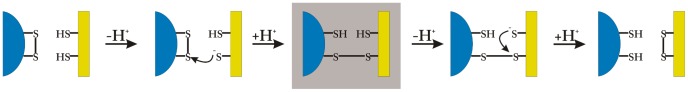
Basic mechanism for disulfide oxidation catalyzed by a thioredoxin-fold domain. (Thioredoxin domain shown in blue). The mixed disulfide intermediate has been highlighted in grey. Note that, unlike the mechanism of disulfide reduction shown in Figure 2, resolution of the intermediate occurs through attack of a thiolate group of the substrate. Actually, attack of the thiolate from the catalyst (as in Figure 2) must be prevented since it would revert the substrate to the initial reduced state.

The processes of thioredoxin-domain catalyzed reduction, formation (oxidation) and reshuffling of disulfide bonds are all dependent on the active-site CXXC motif. Reduction (see Figure 2) starts with the reduced enzyme and involves the nucleophilic attack of the thiolate form of the amino-terminal cysteine on the disulfide bridge of the substrate [34], [35]. The mixed-disulfide thus formed is resolved by the nucleophilic attack of the carbonyl-terminal cysteine. Oxidation, on the other hand (see Figure 3), involves a nucleophilic attack of the substrate on the disulfide bridge of the oxidized enzyme and the mixed-disulfide intermediate is resolved by attack from the free cysteine (in the thiolate form) of the substrate [36]. It is relevant that the oxidation and reduction processes involve opposite chemical changes in the substrate (break-up and formation of disulfide bridges) as well as different mechanisms for the resolution of the mixed-disulfide intermediate. Furthermore, the two processes may be expected to be linked to different values of the redox potential (as suggested by the redox potentials of thioredoxin and PDI: see Figure 6 in Hatahet & Ruddock [36]) and, as it has been extensively discussed in the literature, they likely have different molecular requirements in terms of the conformational changes during catalysis, the stability of cysteine thiolates and the modulation of the pK values of the catalytic groups [33], [36]–[39]. Clearly, disulfide reduction and catalysis of oxidative folding (involving formation of disulfide bridges) may be expected to strongly trade-off.

Contrary to disulfide reduction and formation (Figures 2 and 3), disulfide-bridge reshuffling in misfolded proteins to yield the correctly folded state does not involve a net change in the oxidation state of the substrate and could in principle occur through cycles of catalyzed reduction/oxidation [36], [40]. Alternatively, the initial attack of the enzyme on a substrate disulfide bridge may yield a free cysteine that could attack another disulfide bridge thus starting a cascade of disulfide-bond rearrangments leading to the most stable configuration [36], [40].

The specific protein system we use in this work is *E. coli* thioredoxin, an enzyme involved in multiple reduction processes in vivo [35], [41] which, besides this primary (i.e. reductase) activity is able to catalyze, albeit with very low efficiency, disulfide-bridge-linked protein folding processes [42], [43] (promiscuous activities of *E. coli* thioredoxin). We apply the approach proposed (steps 1–3 above) to *E. coli* thioredoxin with the catalysis of oxidative folding as the promiscuous activity. Nevertheless, the variants thus obtained are also tested for the disulfide reshuffling activity.

## Results/Discussion

### Statistical coupling analysis of the emergence of folding catalysis in the thioredoxin fold

To find a set of positions likely linked to the emergence of disulfide-bridge-linked folding functions in the thioredoxin fold, we have used statistical coupling analysis (SCA) which works by comparing the amino acid distributions at different positions in a multiple sequence alignment (MSA) with the corresponding ones in a given sub-alignment [24]. To apply SCA to the study of primary/promiscuous activity modulation we propose selecting the sub-alignment on the basis of a function-related criterion related with the promiscuous activity. We thus start with a MSA derived from a sequence-database search using the *E. coli* thioredoxin sequence as query and select as sub-aligment those sequences belonging to thioredoxin-fold domains in proteins involved in protein folding in vivo. Actually, this selection step is made straightforward by the fact that these domains contain the active-site CGHC sequence, while thioredoxin reductases contain the CGPC sequence. In fact, the P34H mutation on the *E. coli* thioredoxin background has been shown to enhance significantly its “PDI-like” promiscuous activities [43]. The MSA we have used contains indeed a significant number of sequences with a histidine at position 34 (*E. coli* thioredoxin numbering) that, in most cases, belong to thioredoxin-fold domains of eukaryotic PDI's. We thus obtained the statistical free-energies using the P→H substitution as the perturbation at position 34 (see Methods for details) and we expect these values to reveal networks of residues related with the emergence of the protein folding activities in the thioredoxin fold.

**Figure 4:**
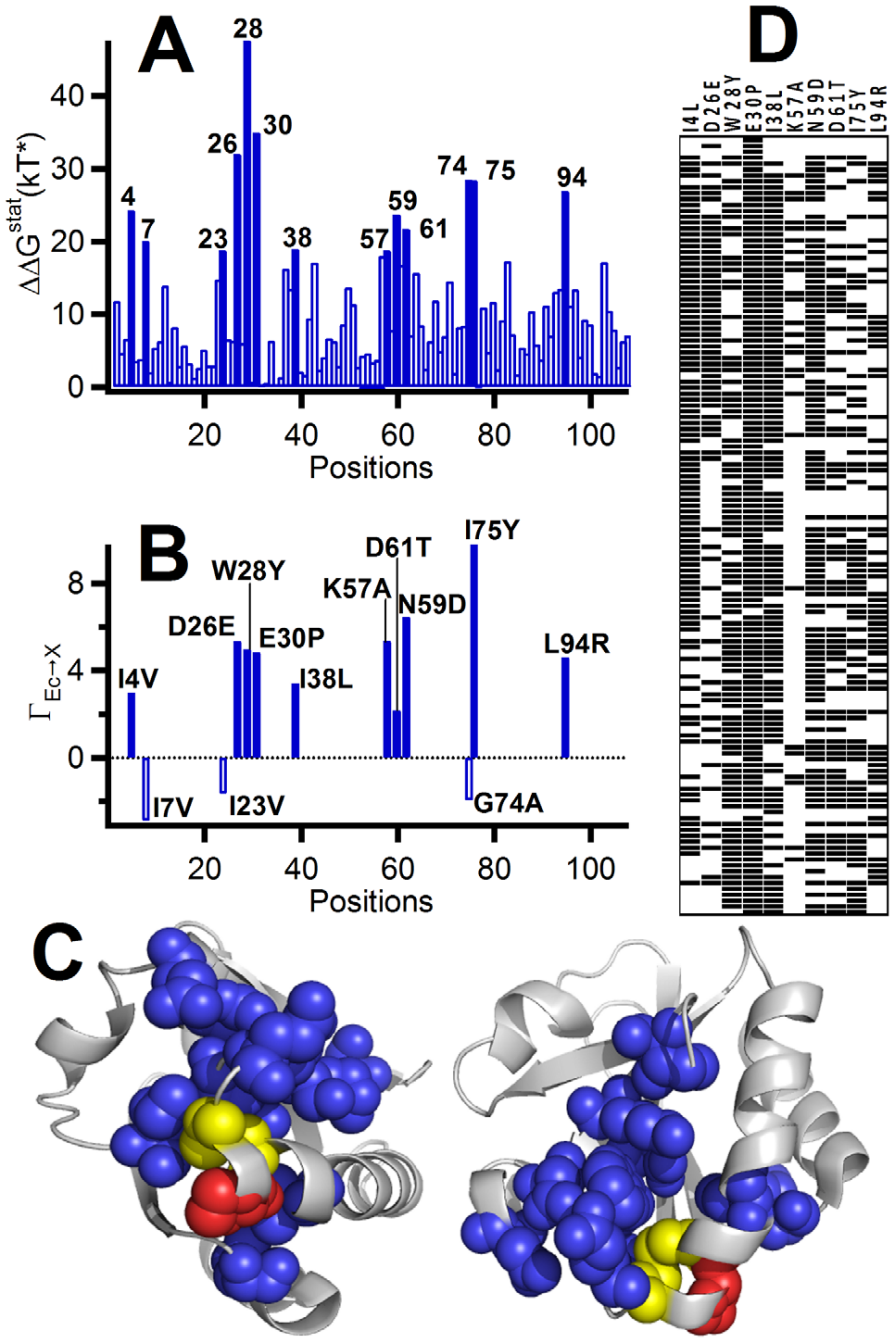
Statistical coupling analysis of the emergence of folding catalysis activities in thioredoxin domains. (A) Statistical free energies for the coupling of position 34 with all the other positions (*E. coli* thioredoxin numbering is used). These values have been calculated using as perturbation the signature P34H mutation. The 13 positions with the highest values for the coupling energies are labeled. (B) Determination of ten favored mutations at the positions selected in step 1 (see text for details and for the definition of the Γ_Ec→X_ function. (C) Mapping of the 10 positions selected for mutation (panel B) on the *E. coli* thioredoxin 3D structure (blue). The active site disulfide bridge is shown in yellow and the proline at position 34 is shown in red. (D) Occurrence of the 10 mutations selected (panel B) in the MSA sequences that include a histidine at position 34. Most of these sequences belong to eukaryotic protein disulfide isomerases. Note that many different combinations of these mutations are actually found in extant PDIs.

However, SCA is based upon the perturbation of the 20 amino acid distribution at each position and actually provides a list of coevolving positions (see Figure 4A), while we are interested in specific mutations at these positions. Therefore, we included an additional layer of statistical analysis. For each given position, we considered the mutation from the amino acid present in *E. coli* thioredoxin (the “Ec” aminoacid) to the amino acid “X” defined as the amino acid different from “Ec” that has the highest frequency (largest number of occurrences in the sequence alignment) when there is a histidine at position 34. Then, for each of the 13 positions with the highest coupling free energies from the SCA analysis (Figure 4A), we calculated the following score:
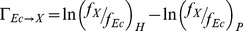
(1)where f is the frequency of occurrence of the amino acids in the sequence alignments and subscripts “H” and “P” refer to the condition for the calculation of the frequencies (histidine or proline at position 34, respectively). Large positive values for the score indicate that the P34H substitution shifts the statistics strongly towards amino acid X. We retained for experimental analysis the 10 positions (and the corresponding Ec→X mutations) for which the score was positive (see Figure 4B): I4V, D26E, W28Y, E30P, I38L, K57A, N59D, D61T, I75Y, L94R. It is important to note that the 10 positions selected form a well-defined, connected network surrounding the active site (see Figure 4C), a fact fully consistent with their likely role in the development of the new functions related with protein folding catalysis. We also wish to emphasize at this point that the immediate purpose of this work (see section below) is to probe the interplay between primary and promiscuous activities in the mutational space defined by the 10 mutations selected. Specifically, the derivation of a molecular-level picture of what each of these mutations is doing (a task that would require extensive structural work due to the potential non-additivity of the mutation effects), is beyond the scope of this work.

### Combinatorial library analysis and partial least-squares reconstruction of the full mutational space

A simple visual examination (see Figure 4D) of the sequences of the MSA used that include histidine at position 34 (most of them belonging to eukaryotic PDI's) shows that different combinations of the 10 mutations selected occur in extant PDI's. We conclude that natural selection does efficiently explore the mutational space defined by combinations of the 10 mutations. To assess how the interplay between the primary and promiscuous activities is modulated in this mutational space, we prepared a combinatorial library spanning the 10 mutations (i.e., 2^10^ = 1024 variants) on the P34H background and determined the reductase and the catalysis of oxidative folding activities for 29 randomly selected variants. The primary activity has been probed by following the standard reduction assay for DTT-reduced thioredoxin which uses insulin as a model substrate (see Methods for details). Clearly, this assay reflects the reduction process of Figure 2. The catalysis of oxidative folding (promiscuous activity in *E. coli* thioredoxin) has been assessed using fully reduced ribonuclease A as substrate (see Methods for details). This assay might include some contribution from the reshuffling process since the first disulfide bridges formed need not be the correct ones. Nevertheless, it is expected to probe mainly the oxidation pathway (Figure 3), an expectation that will be supported by the results reported here.

**Figure 5:**
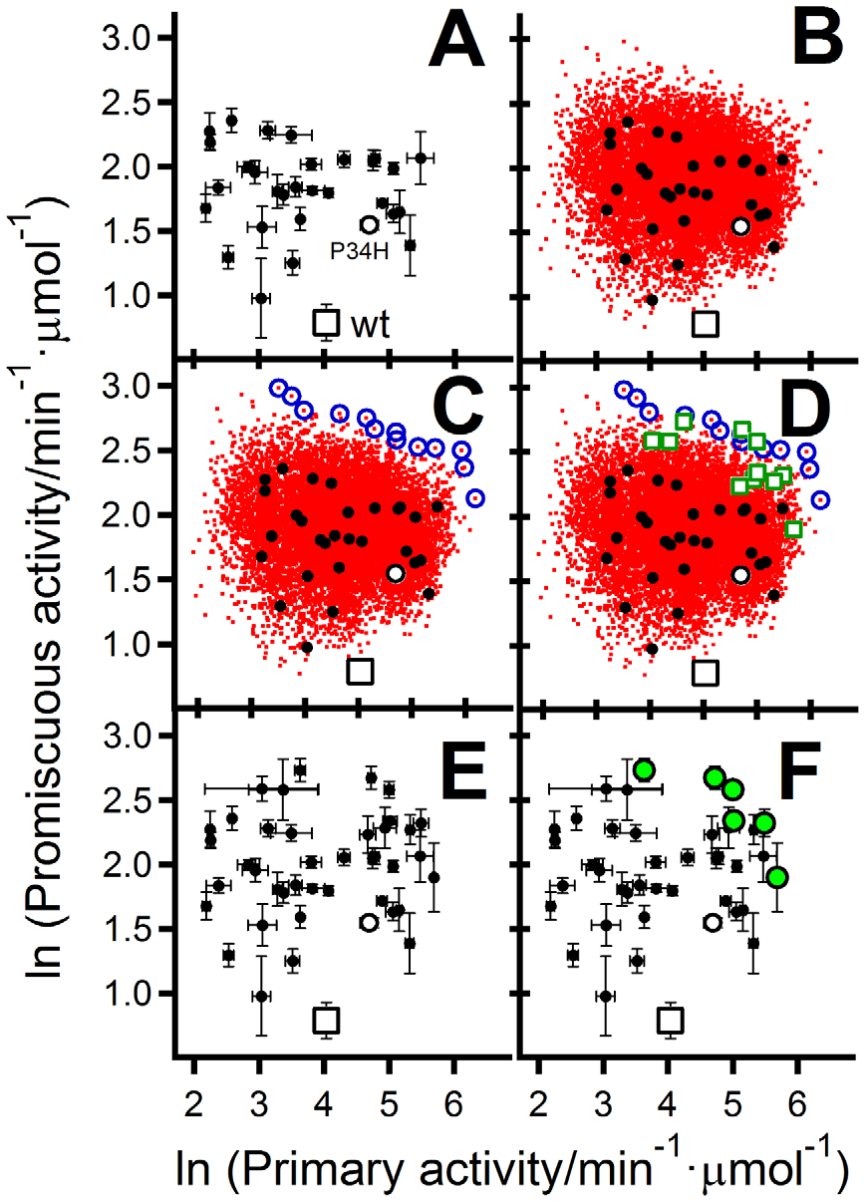
Approximation to the Pareto set of optimum primary/promiscuous activities. (A) Values of the primary and promiscuous activities for 29 variants randomly selected from a combinatorial library based on the 10 mutations derived from SCA analysis (see text and Figure 4). Values for the wild-type thioredoxin from *E. coli* and the background P34H variant are also included. Note that logarithms of activities are used here in and also in all the other panels of this figure. (B) Partial-least-squares (PLS) reconstruction of the data for the whole combinatorial library (small red data points). The experimental data of panel A (used as a basis for the reconstruction) are also shown, although, for the sake clarity we have omitted the errors bars here (as well as in panels C and D). The reconstructed data (red points) are actually derived from 20 bootstrapping replicas (see Methods for details). (C) Prediction of the Pareto set from the PLS-reconstructed data. The 11 variants belonging to this predicted Pareto set are shown with blue circles. (D) The actual experimental activity values for the 11 variants are shown (open green squares). (E) Expanded experimental variant set including the original 29 variants (panel A), the wild-type thioredoxin from *E. coli*, the P34H background variant and the 11 variants added as a result of PLS-reconstruction/Pareto-set-prediction (panels C and D). (F) The actual Pareto set of the expanded variant set is shown (green data points).

Most of the 29 variants screened show increased levels of the promiscuous activity with respect to both the background P34H variant and wt *E. coli* thioredoxin (Figure 5A). This result is consistent with the proposed role of the selected mutations in the emergence of the protein folding activities of the thioredoxin fold. What may perhaps be surprising, however, is that some of the variants also show an increased level of the primary activity, indicating the possibility of the simultaneous enhancement of the primary and promiscuous functions.

In order to assess the full range of function modulation achieved by the combinatorial library we have carried out a fit of the experimental data based on the equation:
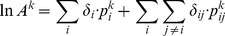
(2)followed by a reconstruction of the entire library using the values of the fitted parameters. The meaning of the symbols in equation 2 is as follows: A^k^ is the dependent variable (activity) with k being a label that identifies the type of activity (i.e., k = “primary” or k = “promiscuous”) = 1; δ_i_ is an independent variable that may take the values 0 or 1, corresponding to the absence o presence of the mutations at position i; p_i_^k^ is a measure of the effect of the mutation at position on the activity A^k^; δ_ij_ = δ_i_·δ_j_ is an independent variable that takes a value of 1 when mutations at positions i and j occur simultaneously (and takes a value of zero otherwise); p_ij_^k^ is a measure of the effect of the coupling between mutations at positions i and j on the activity k. Equation 2 embodies a comprehensive model that includes the effects of individual mutations (p_i_^k^ values) as well as the possibility that mutation effects are non-additive (p_ij_^k^≠0). It involves, however, 110 fitting parameters (10 p_i_^k^ parameters and 45 p_ij_^k^ parameters for each of the two activities), while the number of experimental values to be fitted is only 58 (i.e., the values of the two activities –primary and promiscuous- for the 29 library variants studied). Having more fitting parameters than dependent variable values is a common occurrence in chemometrics, often addressed using partial least-squares [27], [28] (PLS), a dimensionality reduction approach akin to principal component analysis. Indeed, the widespread usefulness of the PLS approach is often credited to its ability to handle a large number of independent variables (i.e., fitting parameters) (see chapter 7 in Livingstone [44]. PLS thus uses latent variables (latent vectors): orthogonal combinations of the original variables that explain most of the variance in the original independent variable set and are also constructed to maximize their covariance with the dependent variables. The original variables may then survive the PLS dimensionality reduction, but they are combined in a few relevant latent vectors. In the case of interest here, once a PLS fit of equation 2 to the experimental data for the 29 variants studied (Figure 5A) has been performed (see Methods for details), it is straightforward to calculate the expected primary and promiscuous activity data for the whole library of 1024 variants. Of course, there remain two important issues related to the assessment of the uncertainty associated to such full-library reconstruction and to its experimental validation. To assess reconstruction uncertainty, we have used a bootstrapping approach involving PLS fits to replica sets obtained by randomly re-sampling from the original experimental set (see Methods for details). Full-library reconstructions resulting from the PLS analyses of 20 such replicas are given in Figure 5B. They clearly suggest that the mutation set derived from the statistical coupling analysis potential has a huge potential for modulating both, the primary and promiscuous activities. Experimental validation of the reconstruction (based on experimental measurements on the predicted Pareto set of optimal variants) is described in the following section.

### Approximation to the Pareto set of variants with optimal primary/promiscuous activities

We derived an “optimistic” prediction of the Pareto set of optimal primary/promiscuous activities (Figure 5C) as the set of non-dominated solutions in the ensemble of reconstructions shown in Figure 5B. The 11 variants in this predicted set were prepared and their activities determined experimentally. There is an excellent qualitative agreement between prediction an experiment, in the sense that, for all the 11 variants, increased levels of both activities were found (Figure 5D). This agreement validates the reconstruction carried out on the basis of the PLS analysis of the 29-variants set.

**Figure 6:**
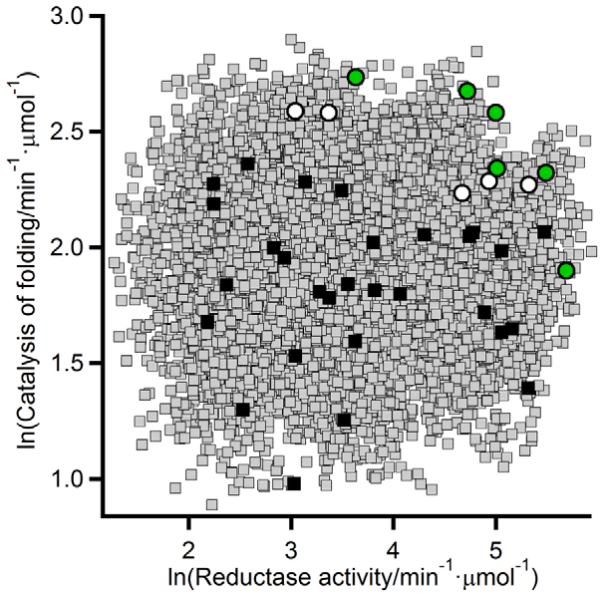
Full-library partial-least-squares reconstructions of the reductase/catalyis-of-oxidative-folding data based upon the expanded 40-variant set. The black squares represent the first-round 29-variant set and the circles represent the 11 variants added as a result of the experimental validation of the Pareto prediction shown in Figure 5C (green data points are used here for the Pareto set). Error bars have been omitted for clarity, but they are shown in Figures 5E and 5F. The reconstructed data (grey squares) are derived from 20 bootstrapping replicas of the 40-variant data. Note that the PLS reconstructions shown here are based on the expanded 40-variant set, while those of Figure 5B were based on the first-round 29-variant set. The full-library reconstructions shown here suggest only small enhancements over the 40-variant Pareto set would be obtained in additional screening rounds and, further, they support that the 40-variant Pareto set is already close to the full-library Pareto set.

It is relevant to note at this point that the PLS-reconstruction/Pareto-prediction analysis leads to an expansion of our experimental variant set (from 29 variants to 40 variants) but in a manner that is not random. Actually, the 11 variants added to the experimental set allow us to move in the space of primary/promiscuous activities in the general direction of the simultaneous enhancement of both activities, as is visually apparent in Figures 5E and 5F. The Pareto set from the experimental data for the 29+11 = 40 variant set (Figures 5E and 5F) is still only an approximation to the Pareto set for the whole library, since additional cycles of PLS-reconstruction/Pareto-prediction could in principle lead to further enhancements in both activities. However, PLS-reconstruction starting with the 40-variant experimental data set suggests that additional improvements are expected to be small (see Figure 6), supporting that 40-variant Pareto set is likely close to the Pareto set of the full library. Furthermore, the main result of the analysis is already apparent with the 40-variant set: *E. coli* thioredoxin, as well as the background P34H variant for library construction, does not belong to the Pareto set and, therefore, simultaneous enhancement of the primary and promiscuous activities is feasible (and has been experimentally achieved: Figures 5E and 5F). Note that, in addition to the targeted simultaneous enhancement (implying enhanced levels for both activities), the experimental data set (as well as the PLS reconstructions of the full combinatorial library) indicates that the two activities can be modulated in an independent-like manner and includes “specialist” variants with a high level for one activity and low value for the other.

### How large are the primary/promiscuous activity modulations achieved?

**Figure 7:**
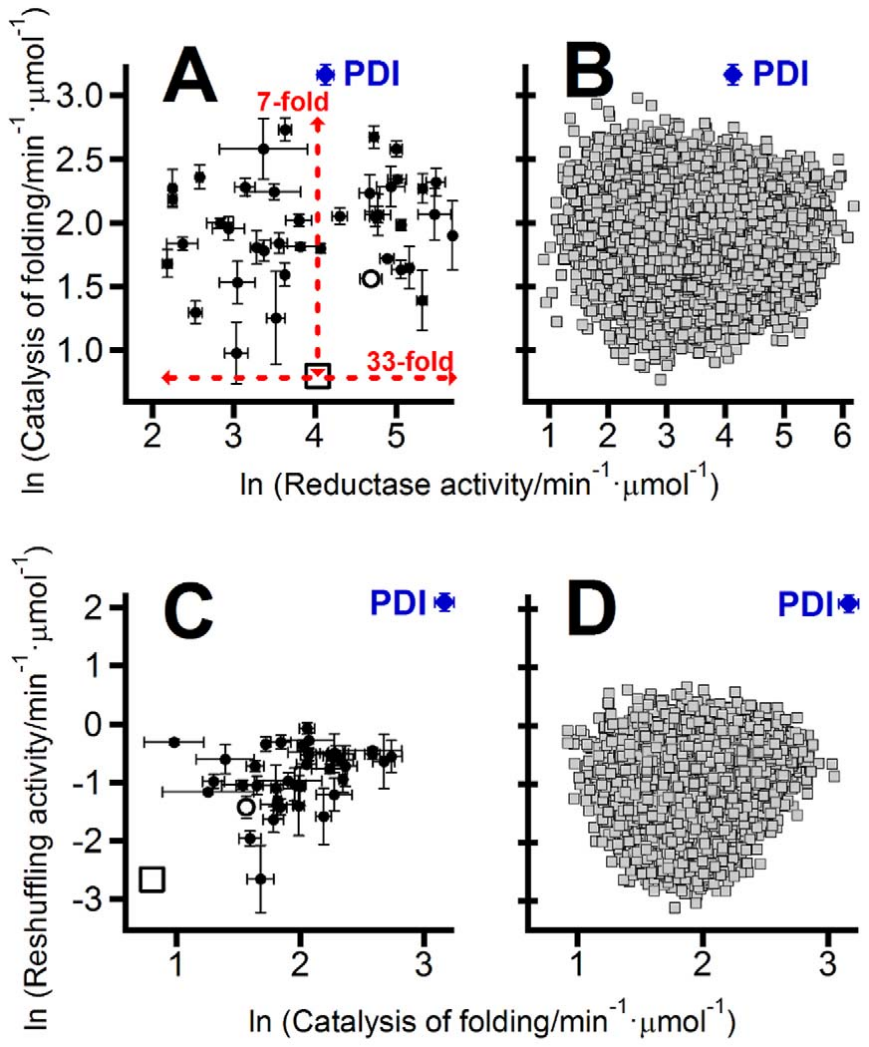
Assessment of the modulation ranges achieved for the primary and promiscuous activities. (A) Experimental reductase and catalysis of oxidative folding data for the expanded 40-variant set, the wild-type thioredoxin from *E. coli* and the background P34H variant. The meaning of symbols is as in Figure 5A. The approximate modulation ranges achieved are indicated with arrowed red lines. Data of bovine protein disulfide isomerase (blue data point labeled PDI) are also included to provide an evolutionary relevant scale for comparison. Note that specific activity data for PDI are given per molar concentration of active thioredoxin domain. (B) Comparison of the PDI experimental data with the partial least squares reconstruction of the reductase/catalysis-of-oxidative-folding data for the whole combinatorial library. The reconstructed data (grey squares) are actually derived from 20 bootstrapping replicas. (C) Experimental disulfide-reshuffling activity and catalysis of oxidative folding data for the expanded variant set, the wild-type thioredoxin from *E. coli* and the background P34H variant. Data of bovine PDI are also included to provide an evolutionary relevant scale for comparison. (D) Comparison of the PDI experimental data with the partial least squares reconstruction of the reshuffling/catalysis-of-oxidative-folding data for the whole combinatorial library. The reconstructed data (grey squares) are actually derived from 20 bootstrapping replicas.

Figure 7A highlights the modulation ranges experimentally achieved for the reductase and catalysis of oxidative folding activities (about 33-fold and 7-fold, respectively). One important issue is whether these ranges (in particular that of the promiscuous activity) are to be considered large or small. The answer to this question depends largely on the relevant context. Certainly, the modulation ranges we have found are much smaller than those reported in some protein design studies (consider, for instance, the 200-fold enhancement in engineered Kemp eliminase activity reported by Baker, Tawfik and coworkers [45]) and the ranges typically considered relevant in a biotechnological application context. It is important to note, however, that the approach we have used is aimed at assessing the patterns of primary/promiscuous activity modulation in the mutational space actually explored by natural evolution when recruiting the promiscuous activity for a new function. That is, the mutations included in our combinatorial library are those expected to be associated to the emergence of folding catalysis in the thioredoxin scaffold during the course of natural evolution. If the approach is successful, promiscuous activities approaching the levels of natural thioredoxin-scaffold folding catalysts should be reached. In an evolutionary/biological context, therefore, the promiscuous activity modulation achieved should be compared with the evolutionary significant modulation range estimated on the basis of activity data for a protein disulfide isomerase. Experimental data for bovine PDI are included in Figure 7 and indeed show an acceptable level of congruence with the Pareto set for the 40-variant variant set (Figure 7A) as well as with the corresponding PLS reconstructions (Figure 7B).

Clearly, the modulation range achieved for the catalysis of oxidative folding is significant from a biological/evolutionary point of view. Interestingly, this does not appear to hold for the other folding-related activity of thioredoxin domains: the disulfide-reshuffling activity responsible for the rescue of misfolded proteins with incorrect disulfide bridges. Figure 7C is a plot of reshuffling activity (measured using disulfide-scrambled ribonuclease A as substrate: see Methods for details) versus catalysis of oxidative folding, including the 40-variant set, wild-type thioredoxin from *E. coli*, the P34H background variant and bovine PDI. Figure 7D is a similar plot including the PLS reconstructions based on the experimental data of Figure 7C. These two plots suggest that the combinatorial library used (based on the mutation set derived from SCA analysis: Figure 4) spans the evolutionary relevant range for the catalysis of oxidative folding, but not the corresponding range for the reshuffling activity. This result is actually consistent with some known features of the structure-function relationship in protein disulfide isomerases. PDIs have a multidomain structure usually described [46] in terms of four distinct domains (a b b′ a′), two of which (the a and a′ domains) display the thioredoxin-fold structure and the CXXC active site motif responsible for the catalysis of disulfide-linked process. The isolated a and a′ domains have been shown to introduce efficiently disulfide bridges into proteins [47], while additional domains are required for efficient catalysis of disulfide bond reshuffling in folded proteins [48]–[50], perhaps because the “inactive” b and b′ domains play a role in facilitating steps that involve difficult conformational changes [48]. Obviously, this “multidomain” effect cannot be reproduced by engineering based on a single-domain thrioredoxin scaffold.

### The shape of the Pareto set in the primary/promiscuous activity plot is consistent with the conformational diversity hypothesis

**Figure 8:**
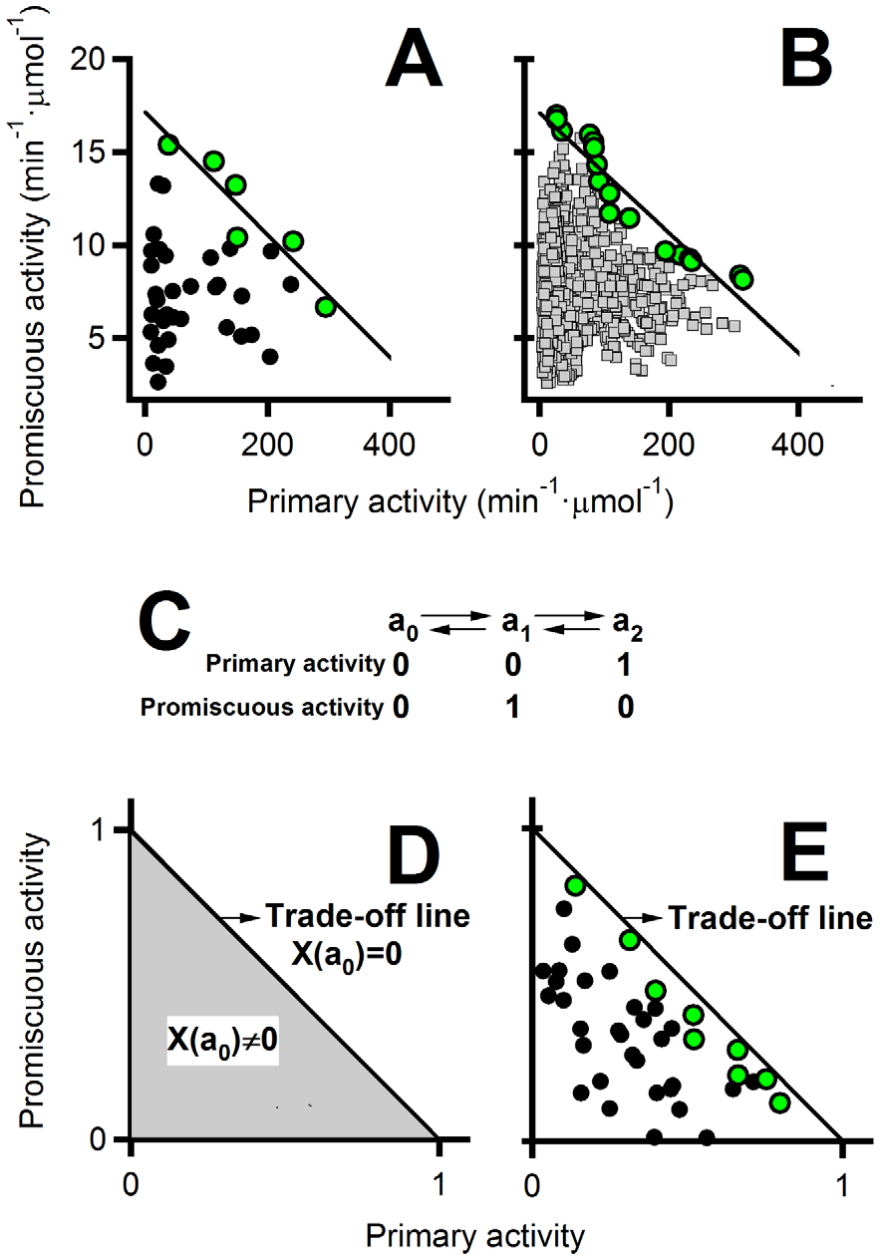
The shape of the primary/promiscuous Pareto set is consistent with the conformational diversity hypothesis. (A) Values of the reductase and catalysis of oxidative folding activities for the 40-variant experimental set. The Pareto set is shown with green data points. This plot is analogous to that in Figure 5F but linear activity scales (instead of logarithms) are used here and error bars have been omitted for the sake of clarity. The line represents the least-squares fit to the Pareto set data. (B) A partial least squares reconstruction of the data for the whole combinatorial library based on the experimental data of panel A. The data shown correspond to a single bootstrapping replica; however, other replicas show the same general pattern The Pareto set (green points) and the corresponding linear fit are shown. (C) Simple conformational diversity model used in the simulation summarized in panels D and E. Conformation a_0_ is not active, while conformations a_1_ and a_2_ are responsible for the promiscuous and primary activities, respectively. (D) Relevant regions in the primary/promiscuous activity diagram according to the model shown in panel C. Optimal situations correspond to a zero mol fraction of the inactive conformation and define a *trade-off line* in the plot. Sub-optimal situations correspond to a mol fraction of a_0_ higher than zero and are located in a triangular-shaped region below the trade-off line. (E) Stochastic simulation of 40-variant set based on the model shown in panel C. The Pareto set (green data points) approaches the trade-off line.

The primary/promiscuous plots presented so far (Figures 5, 6 and 7), employ logarithmic activity scales in order to emphasize the order of magnitude of the modulations achieved. However, using linear scales in these plots (Figures 8A and 8B) reveals a surprisingly simple pattern: a linear-like Pareto set and a roughly triangular shape for the “cloud” of experimental data points below the Pareto set. This pattern is robust, being observed in the 40-variants data set and in the PLS reconstructions of the full combinatorial library. Note also that the observed pattern implies that essentially all the experimental data points are at or below a line connecting the expected maximum values for the primary and promiscuous activities and, therefore, that the experimental data points populate an area in the primary/promiscuous activity plot which is about half the maximum area accessible. The probability of this happening by chance if there is no correlation between the primary and promiscuous activities is on the order of (1/2)∧NDP being NDP the number of experimental data points. This gives a negligible probability for NDP = 1024 even for NDP = 40. Finally, as we have already pointed out, the second-round of the library screening process was sharply focused to the Pareto set and that, as a result, the Pareto set of the 40-varaints experimental set is likely to be close to the Pareto set of the full library. We conclude from all this reasoning that the simple experimental pattern in Figures 8A and 8B is robust and is unlikely to have arisen by chance. It is natural then to seek a simple interpretation for such a simple, but intriguing pattern. As we elaborate below one simple explanation is provided by the so-called conformational diversity hypothesis.

The conformational diversity hypothesis posits native proteins may exist in solution as different conformations in equilibrium and provides a plausible structural rationale for the existence of protein promiscuity [5], [51], [52]. In very simple terms, the most populated (i.e., dominant) conformation is responsible for the primary activity while alternative, low-population conformations perform the promiscuous activities. Mutations can shift the equilibria between the different conformations and thus modulate the balance between the primary and promiscuous activities. A linear-like Pareto set could thus be explained in terms of two optimal conformations, each being responsible for catalyzing efficiently only one of the activities. For instance, one conformation would achieve molecular optimization for the substrate reduction process (Figure 2) when the active-site disulfide is reduced, while an alternative conformation would achieve optimization for substrate oxidation (Figure 3) when the active-site disulfide is oxidized. Obviously, data points below the Pareto set would correspond to significant population of other conformations that are suboptimal in terms of activity. This interpretation is clarified below with a simple illustrative example.

Consider three protein conformations: **a_0_**: a conformation with no activity; **a_1_**: the conformation responsible for the primary activity; **a_2_**: the conformation responsible for the promiscuous activity. The mol fractions of the three conformations must add up to unity:
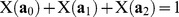
(3)Mutations may change these mol fractions and, obviously, the optimal primary/promiscuous activity situations will be achieved when X(**a_0_**) = 0 and,
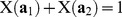
(4)Since activities should be proportional to the corresponding mol fractions, equation 4 defines a straight line in a plot of promiscuous versus primary activity. We refer to this line as the “trade-off line”. In the same plot, suboptimal situations in which X(**a_0_**)≠0 will necessarily be represented by points in a triangular area defined by the trade-off line and the plot axes (see Figure 8D). Certainly, the plot in Figure 8D (showing the optimal trade-off line and a shaded triangular region corresponding to suboptimal situations) is an idealized representation. In practice, we must consider the possibility that the mutations used are unable to completely shift the equilibria towards the active conformations (i.e., they might be unable make the mole fraction of the inactive conformation strictly equal to zero). To provide an illustration of this situation, we have carried out a stochastic simulation of a 40-variant data set, assuming that conformation populations are proportional to statistical weights derived from flat distributions. That is, the mol fraction of a given conformation a_i_ is given by w_i_/Σw_i_ where w_i_ is its statistical weight (derived from a random number generator in the [0,1] interval) and Σw_i_ is the sum of the statistical weights for all the conformations. The result of this simulation (Figures 8E) is a roughly triangular-shaped cloud of data points with a linear-like Pareto set that approaches the trade-off line.

**Figure 9:**
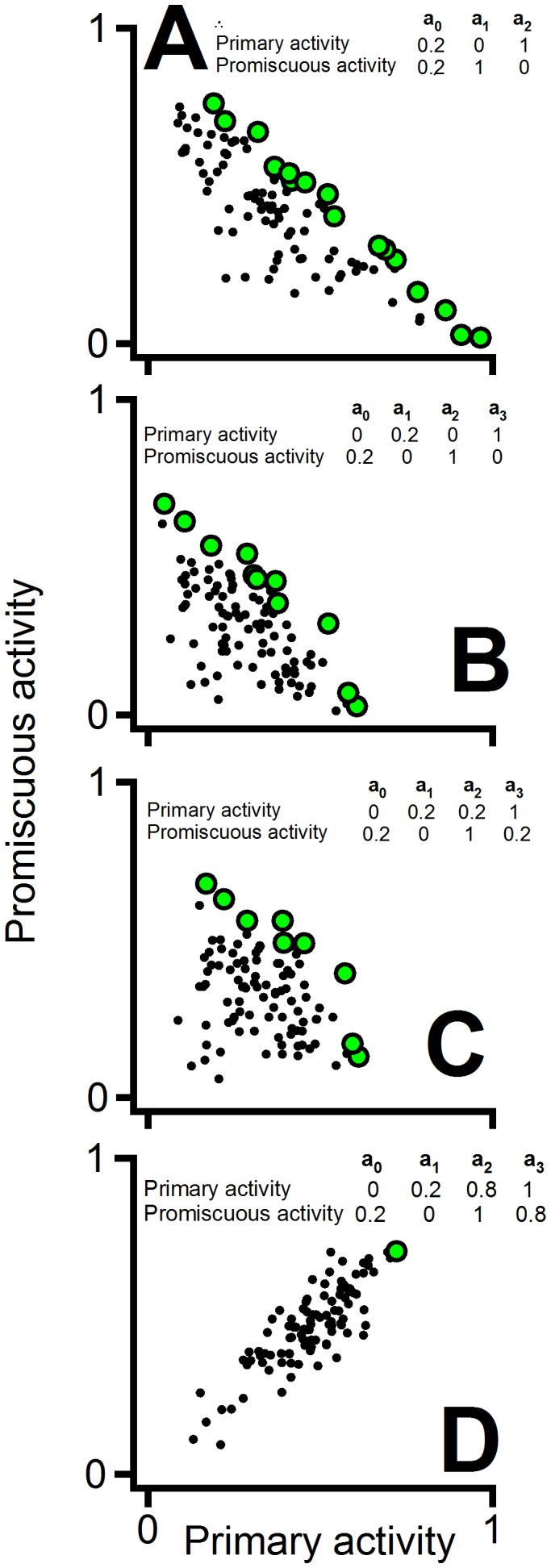
Stochastic simulations of primary/promiscuous activities based on different conformational diversity models. (A) Simulation based on a 3-conformation model identical to that used in the simulation of Figure 8E, except that a low (but different from zero) level of primary and promiscuous activities for the suboptimal conformation (**a_0_**) is assumed. Here, as well as in the other panels, the Pareto set is shown with green circles. (B) Simulation based on a 4-conformation model with two suboptimal conformations. (C) Same as in (B), except that each one of the optimal conformations (**a_2_** and **a_3_**) has a low level of the alternative activity. (D) Same as in (C), but assuming that the optimal conformations show high level of both activities. The models used in (A), (B) and (C) include the existence of trade-offs between the optimal conformations and yield roughly linear Pareto sets with a significant number of data points below the Pareto set due to the presence of suboptimal conformations. This is actually the experimental pattern we have found for the catalysis-of-oxidative-folding/reductase activities (Figure 8A). The model used in (D) does *not* include trade-offs between the optimal conformations (i.e., **a_2_** and **a_3_** efficiently catalyze both, the primary and the promiscuous processes) and the pattern is completely different: a significant correlation between the two activities and a very small Pareto set are observed.

For simplicity and illustration, the simulations included in Figure 8 assume that there is only one sub-optimal conformation and that it has zero primary and promiscuous activity levels. It is important to note, however, that the general result of the simulations is robust and it is obtained with different conformation models (see Figures 9A–C) including several sub-optimal conformations with non-zero activity levels. Apparently, all that is required for a linear-like Pareto set to be obtained in these simulations is a model with two optimal conformations with quite different capabilities to catalyze the primary and promiscuous processes. Actually, if the two optimal conformations are efficient at catalyzing both activities (and, therefore, there are no trade-offs!), the pattern of a linear-like Pareto set with a triangular-like data points cloud is not obtained (see Figure 9D for a representative simulation).

Certainly, the simulations discussed above (Figures 8 and 9) are not meant to be taken as direct evidence in support of the conformational diversity. In fact, obtaining such direct evidence would require extensive structural and dynamic characterization (see, for instance [53] and [54]) which is beyond the scope of this work. Because of this we cannot rule out that other kinds of models (based, for instance, on modeling the mutation effects on the activities) could also explain the experimental results founds. Nevertheless, it is clear that the conformational diversity hypothesis provides a simple, Occam-razor explanation (since modeling of specific mutation effects is not involved) for an equally simple, but otherwise intriguing, experimental modulation pattern.

### Diversity of patterns of primary/promiscuous activity modulation

**Figure 10:**
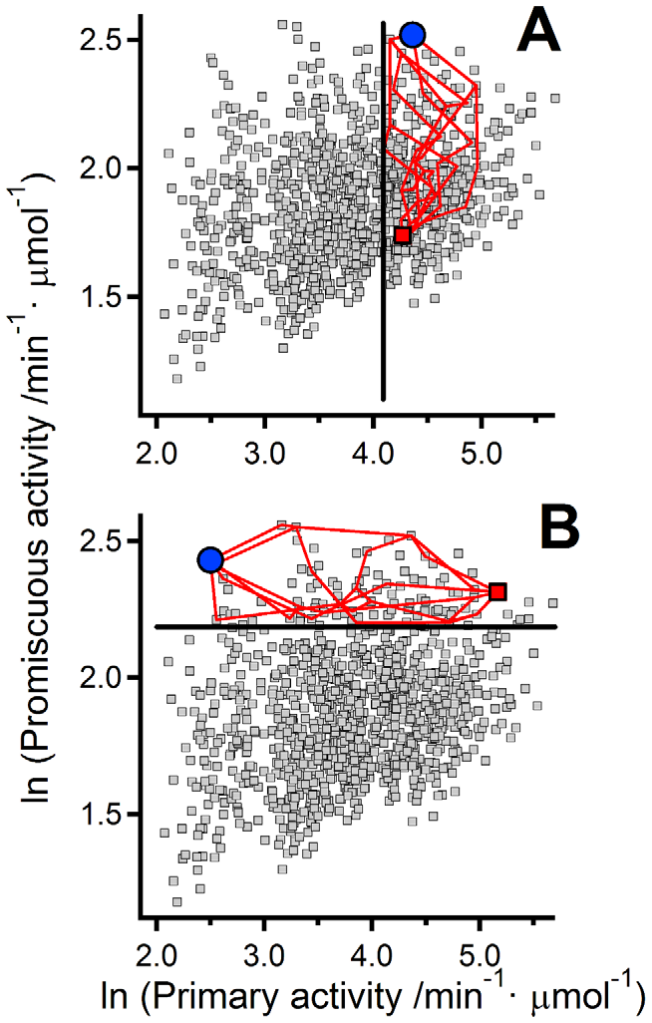
Diversity of mutational paths in the primary/promiscuous activity space. Grey data points represent the results of a PLS reconstruction based on the 40-variant experimental set. Mutational paths (red lines) connecting a starting variant (red data point) and a final variant (blue data point) are shown. (A) The starting variant shows a low level of the promiscuous activity and a comparatively high level of the primary activity. Mutational steps are allowed if promiscuous activity is increased and the primary activity is maintained above a given threshold (shown as a vertical black line). (B) The starting variant shows high level of both, the primary and promiscuous activities. Mutational steps are allowed if the primary activity is decreased and the promiscuous activity is maintained above a given threshold (shown as a horizontal black line).

The simultaneous enhancement of the primary and promiscuous activities we have discussed in the preceding sections is only one aspect (albeit a prominent one) of a general property of the set of mutations derived from the function-based statistical coupling analysis: the potential for originating a multiplicity of mutational paths leading to different types of function modulation patterns. To illustrate the idea (Figure 10) we use one of the full-library reconstructions derived from the PLS analysis of the 40-variants experimental set. Each of the mutational paths shown in Figure 10 has been constructed using the following procedure: a) An initial variant is chosen; b) the variants connected to the chosen one by single mutations are tested for a given activity-related condition; c) one variant among those that pass the test is randomly selected; d) the cycle a-c is repeated until no mutational steps are available.

The paths shown in Figure 10A start with the background variant (i.e., the variant with no mutations) and mutational steps are allowed if promiscuous activity (catalysis of oxidative folding) is increased while the primary activity (reductase) is maintained above a certain threshold. These simulations illustrate the case in which there is selection for enhanced promiscuous activity while maintaining a level of the primary activity that does not compromise fitness. Several paths lead to a variant with increased promiscuous activity and still a significant level of primary activity. Interestingly, PDIs show a significant level of reductase activity (see published work [47], [48], [50] and Figure 7), perhaps because the catalysis of disulfide-linked folding likely involves steps in which incorrect disulfide bridges must be broken up. It is thus tempting to speculate that the no-trade-off paths in Figure 10A illustrate some of the actual function changes taking place in the evolution of these disulfide-linked folding catalysts. It is also interesting that some of the intermediate variants in the paths of Figure 10A have significantly increased levels of both activities, again emphasizing the possibility of the simultaneous enhancement of the primary and promiscuous functions.

The Paths shown in Figure 10B use as starting point a variant with comparatively high values of both, the primary activity and the promiscuous activity (actually, a member of the Pareto set of optimal solutions) and mutational steps are allowed if the primary activity is decreased while the promiscuous activity remains above a given threshold. The paths in Figure 10B lead to a specialist protein with high promiscuous activity and low primary activity. These paths could be viewed as illustrating the molecular changes that could in some cases occur in one of the gene copies arising from the gene duplication event involved in the emergence of a new function. According the so-called balance hypothesis [55], single-gene duplication may actually be harmful because it immediately leads to a very large excess of a given protein, which may be deleterious. If imbalance is associated to an excessive level of the primary function, then the mutational paths in Figure 10B illustrate a potential of the mutational space explored by natural selection to efficiently restore balance.

A final clarification should be made. The mutational paths in the illustrative simulations summarized in Figure 10 have been obtained assuming that all the single-mutation steps can be readily achieved, although some of them cannot be realized with a single-base substitution. However, these amino acid substitutions do occur during natural evolution (involving an intermediate amino acid) as clearly shown by the sequences in Figure 4D. In connection with this, it is important to note that the mutational space we have characterized is very likely a subspace of the full mutational space explored by natural selection in the evolution of disulfide-linked folding catalysts (the latter involving additional positions and several mutations at each position). Obviously, this fact only reinforces our conclusions.

### Concluding remarks

Current views on the relation between primary and promiscuous protein activities are derived to a significant extent from laboratory evolution experiments aimed at enhancing promiscuous functions. Many of these studies have found a decrease (often moderate) in primary activity concomitantly with the increase in promiscuous function, suggesting that the two activities trade-off. In this work, we have introduced an approach to determine how the interplay between the primary and promiscuous activities of a protein is modulated in the mutational space evolutionary linked to the emergence of a new function. Application of this new approach to the emergence of folding catalysts reveals a hitherto unexplored scenario: diverse patterns of primary/promiscuous activity modulation may occur as response to different types of evolutionary pressure, including no-trade-off paths involving the simultaneous enhancement of both activities. Some general remarks related with this result are appropriate:

1. Although admittedly moderate, the simultaneous enhancement we have achieved is in the range expected for modulations that are significant in a biological context, as indicated by the acceptable level of congruence of PDI data with the Pareto set for the 40-variant variant set (Figure 7A) as well as with the corresponding PLS reconstructions (Figure 7B).
2. We have used in this work combinatorial-library screening to probe the mutational interplay between primary and promiscuous activities as assessed by established *in vitro* assays, while the interplay between the two activities *in vivo* may also be determined by additional (non-mutational) factors, such as the redox environment [56]. It must be noted, however, that the mutations included in our library were derived from a coupled correlation analysis (SCA) targeted to the evolutionary emergence of folding catalysis in the thioredoxin scaffold and, further, that combinations of these mutations do enhance this activity in *E. coli* thioredoxin to levels approaching those of the natural folding catalysts (PDI data: see Figure 7A). There can be little doubt, therefore, that the mutational modulations captured by our approach have an evolutionary significance related with new function emergence. In particular, the possibility arises that processes of new function evolution could actually involve no-trade-off mutational paths prior to the gene duplication event.
3. Contrary to naïve intuition, the fact that variants with simultaneously enhanced levels of primary an promiscuous activities can be obtained is not necessarily in conflict with the existence of trade-offs between these two activities, because trade-offs are confined to the Pareto set of optimal primary/promiscuous solutions while most of the variants spanned by the mutational space studied do not belong to this Pareto set.
4. Obviously, the simultaneous enhancement of primary and promiscuous activities relies on the fact that the maximum possible values of the two activities are not realized in the variant used as background of the mutational analysis. The implication is that the background, wild-type protein is not only sub-optimal for its promiscuous activity (as was to be expected), but that it is also sub-optimal for its primary activity. Interestingly, a very recent analysis of the catalytic parameters for several thousand enzymes [57] indicates that most enzymes have moderate catalytic efficiency and supports that primary-activity enhancement should be possible in many cases. The scenario we have described in this work for the mutational interplay between primary and promiscuous activities could then be expected to be widespread, and not limited to the specific type of protein promiscuity (forward/reverse reactions) we have experimentally studied.
5. The partial-least-squares/Pareto-set-prediction protocol we have used provides an efficient computational basis to library screening targeted at the in vitro enhancement of several protein activities. The general result obtained using this protocol will of course depend on the type of library being screened. Libraries focused on the basis of sequence alignment information (such as that used in this work) will likely lead to the comparatively small enhancements that are relevant in a biological context and that may be informative about the evolution of protein function. However, libraries focused on the basis of successful computational design and very large random libraries may lead to the large enhancements that are significant in a biotechnological setting. Therefore, the availability of partial-least-squares/Pareto-set-prediction protocol, together with the general description introduced here for the interrelation between the several activities of a protein and the mutational space explored, should pave the way for the engineering of multi-functional enzymes.

## Methods

### Sequence alignment and Statistical Coupling Analysis

BLAST2 (1996–2003, W. Gish http://blast.wustl.edu) was used to search the TrEMBL sequence database of October-2007 (http://www.ebi.ac.uk/trembl) using the sequence of *E. coli* thioredoxin as query. The resulting sequences were aligned with the query sequence using the Smith-Waterman algorithm and only those with sequence identity with the query of 0.3 or higher were retained for further analysis. We made no further attempt to correct or filter the alignment, since the results obtained from its analysis made clear sense from both, the structural and functional viewpoints (see Figures 4 and 5). Of the1440 sequences in the alignment used, 1264 had a proline at position 34 and 132 had a histidine at that position. Essentially all the sequences with histidine at position 34 belonged to eukaryotes and most of them were actually annotated as protein disulfide isomerases.

Statistical coupling analysis of the sequence alignments based on the P34→H perturbation were performed using homemade programs, but in a manner identical to that described by Lockless and Ranganathan [24]. The robustness of this analysis is supported by the fact that the positions with high values for the statistical coupling energy also rank high in a simple covariance analysis [58] of the sequence alignments (see Figure S1 in Supporting Information).

### Variant library generation and protein purification

The combinatorial library of thioredoxin variant sequences on the P34H background was prepared by using gene assembly mutagenesis as we have previously described [59]. For ease of protein purification, the genes encoded a His_6_ tag at the N-terminal end (i.e., at a position roughly opposite to the active-site region). Purification of the thiredoxin variants, assessment of their purity and concentration measurements, were performed as previously described [59]. Bovine PDI was purchased from Sigma and used without further purification.

### Activity determinations

Reductase activity of the thioredoxin variants was determined at 37°C by a turbidimetric assay of the thioredoxin catalyzed reduction of insulin [60]. Briefly, thioredoxin-variant (or PDI) solutions at pH 6.5 (phosphate buffer 0.1 M) in the presence of 2 mM EDTA and 0.5 mg/mL insulin were prepared. The reactions were initiated by addition of DTT to a 1 mM final concentration and monitored by measuring the absorbance at 650 nm (A_650_) as function of time. Activity is calculated as the maximum value of the change of A_650_ with time, i.e., the maximum value for the derivative dA_650_/dt (see Figures S2 and S3 in Supporting Information for representative examples). Typically, for each variant, 3–4 experiments at different thioredoxin-variant concentrations (within the 0–5 µM range) were carried out and the specific activity values, together with its associated standard errors, were determined from linear fits to the activity versus concentration profiles: see Figure S4 in Supporting Information for representative examples and for further details.

The catalysis of oxidative folding activity was determined by following the recovery of ribonuclease A (RNase A) activity from completely reduced RNase A following the procedure described by Lundström et al. [43]. Briefly, nitrogen-saturated solutions of thioredoxin variants (or PDI) in 0.1 M phosphate buffer pH 7 in the presence of 1 mM EDTA and 100 µM oxidized glutathione were prepared. The reaction was initiated by addition of RNase A from a stock solution to a final concentration of 0.4 mg/mL. After 1 hour incubation at 37°C, the RNase A activity was determined using the standard assay based on the hydrolysis of 2′-3′-cCMP. Typically, for each variant, 4 experiments at different thioredoxin-variant concentrations (within the 0–15 µM range) were performed and the specific activity values, together with its associated standard errors, were determined from linear fits to the recovered RNase A activity versus concentration profiles. Assays for the disulfide reshuffling activity were carried out in the same way, except that disulfide-scrambled RNase A was used and 100 µM reduced glutathione was included in the reaction solution. Fully-reduced and scrambled RNase A were prepared as described by Lündstrom et al. [43] See Figures S5 and S6 in Supporting Information for representative examples of the disulfide-linked folding assays and for further details.

### Partial least-squares analysis

PLS analyses were carried out with the program Unscrambler X from CAMO software using the NIPALS algorithm. In all cases, the dependent variables were the logarithms of the values for the primary and promiscuous activities and were auto-scaled (i.e., they were subjected to mean subtraction followed by division by the standard deviation) prior to the analysis. Leave-one-out cross-validation was used and the number of latent variables retained was the optimum value suggested by the Unscrambler program on the basis of the mean square error of cross-validation. Actually, the PLS analyses were carried with 20 replica sets constructed from the original set through random re-sampling (bootstrapping) and the number of latent variables retained did depend on the replica set used; typical values, however, were on the order of 3–11 (i.e., much smaller than the numbers of dependent and independent variables involved). Illustrative plots experimental versus predicted activities are given in Figure S7 in Supporting Information.

## Supporting Information

Figure S1 **Comparison between the statistical free energies derived from SCA analysis and the results of a simple covariance analysis (σ values).** The values shown correspond to the correlation of position 34 with all other positions in the thioredoxin sequence. The 13 positions with the highest statistical free energy values (see Figure 4A in the main text) are shown here with closed circles.
(TIF)

Figure S2 **Determination of the reductase activity of thioredoxin variants.** Respresentative plots of absorbance at 650 nm versus time for the reduction of insulin catalyzed by thioredoxins. Profiles for the wild-type thioredoxin from *E.coli* and two variants are shown.
(TIF)

Figure S3 **Determination of the reductase activity of thioredoxin variants.** Plots of derivative of absorbance versus time corresponding to the profiles shown in Figure S2. The activity value for each variant at the concentration shown is calculated as the maximum value of dA_650_/dt.
(TIF)

Figure S4 **Determination of the reductase activity of thioredoxin variants.** Specific activity is determined from the slopes of plots of activity (maximum value of dA_650_/dt) versus protein concentration. Several representative examples are shown, including wild-type thioredoxin from *E.coli* and several variants.
(TIF)

Figure S5 **Determination of the catalysis of oxidative folding activity of thioredoxin variants.** Plots of recovered RNase activity from fully reduced protein versus thioredoxin variant concentration. Specific activity is calculated as the slope of these plots. Recovered RNase activity is measured by the initial rate of the change of the absorbance at 288 nm that accompanies the hydrolysis of 2′-3′-cCMP. Several representative examples are shown, including wild-type thioredoxin from *E.coli* and several variants, one of which is the P34H variant used as background for library construction.
(TIF)

Figure S6 **Determination of the disulfide reshuffling activity of thioredoxin variants.** Plots of recovered RNase activity from disulfide-scrambled protein versus thioredoxin variant concentration. Specific activity is calculated as the slope of these plots. Recovered RNase activity is measured by the initial rate of the change of the absorbance at 288 nm that accompanies the hydrolysis of 2′-3′-cCMP. Several representative examples are shown, including wild-type thioredoxin from *E.coli* and several variants, one of which is the P34H variant used as background for library construction.
(TIF)

Figure S7 **Representative examples of partial least squares fits to the 29-variants set.** (Figure 5A). Actually, fits to 4 bootstrapping replicas (color-coded) extracted from that set are shown.
(TIF)
