# Supplementary figures and images for: Probing the Mutational Interplay between Primary and Promiscuous Protein Functions: A Computational-Experimental Approach
Source: PLoS Comput Biol. 2012 Jun 14;8(6):e1002558. doi: 10.1371/journal.pcbi.1002558 (PMC3375227; doi:10.1371/journal.pcbi.1002558)

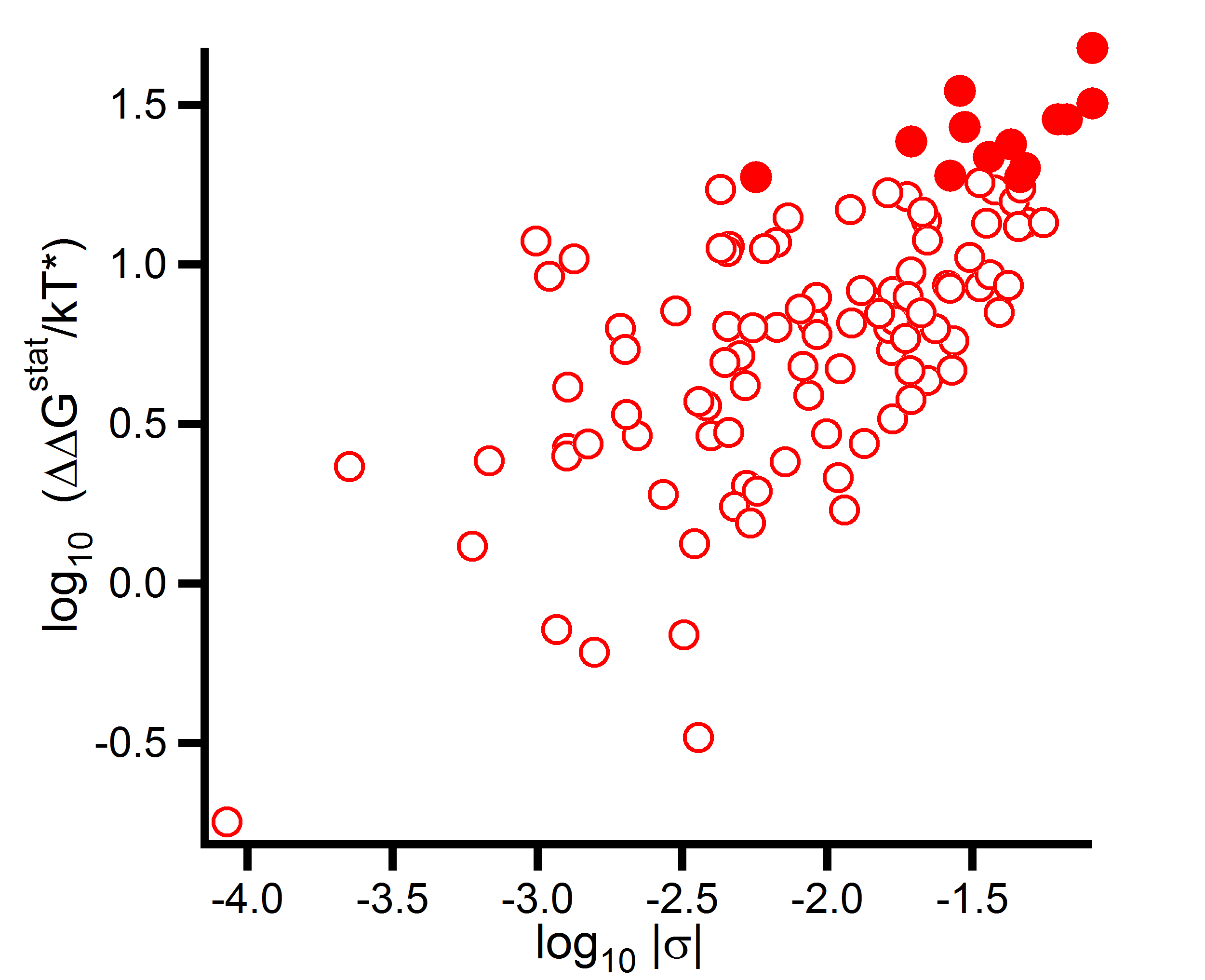

Supplement: Figure S1 — Comparison between the statistical free energies derived from SCA analysis and the results of a simple covariance analysis (σ values). The values shown correspond to the correlation of position 34 with all other positions in the thioredoxin sequence. The 13 positions with the highest statistical free energy values (see Figure 4A in the main text) are shown here with closed circles. (TIF) [file pcbi.1002558.s001.tif]

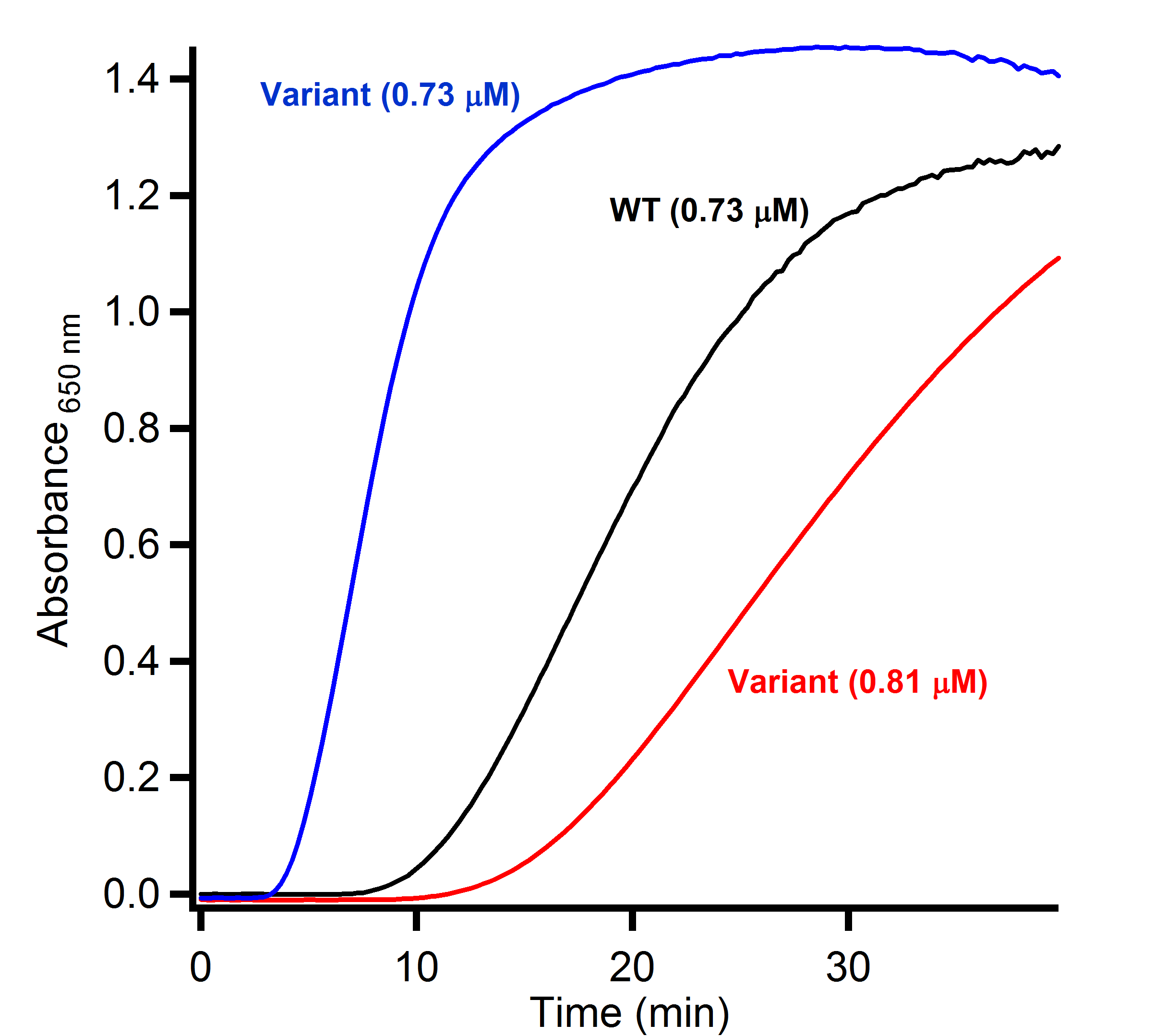

Supplement: Figure S2 — Determination of the reductase activity of thioredoxin variants. Respresentative plots of absorbance at 650 nm versus time for the reduction of insulin catalyzed by thioredoxins. Profiles for the wild-type thioredoxin from E.coli and two variants are shown. (TIF) [file pcbi.1002558.s002.tif]

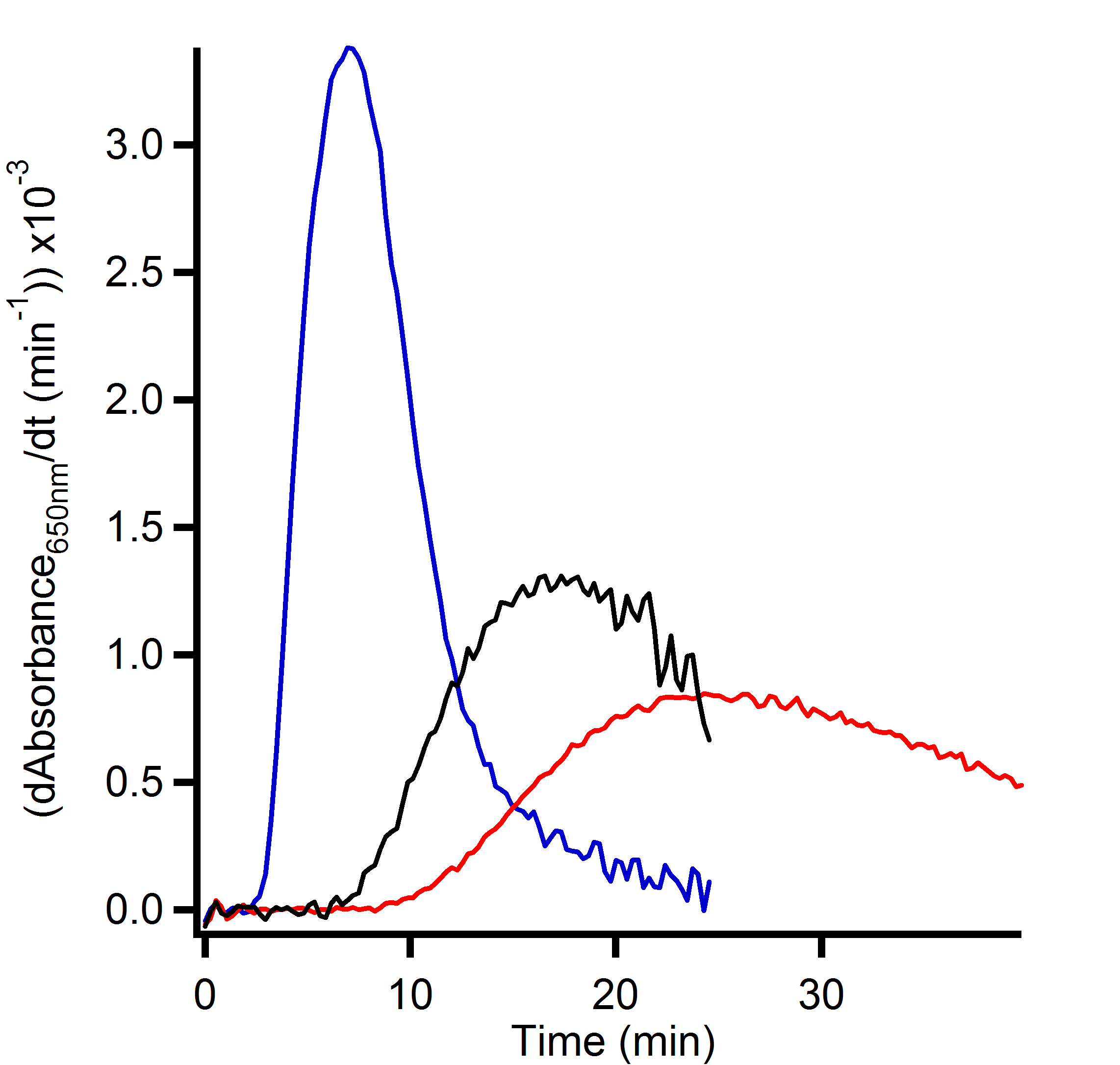

Supplement: Figure S3 — Determination of the reductase activity of thioredoxin variants. Plots of derivative of absorbance versus time corresponding to the profiles shown in Figure S2. The activity value for each variant at the concentration shown is calculated as the maximum value of dA650/dt. (TIF) [file pcbi.1002558.s003.tif]

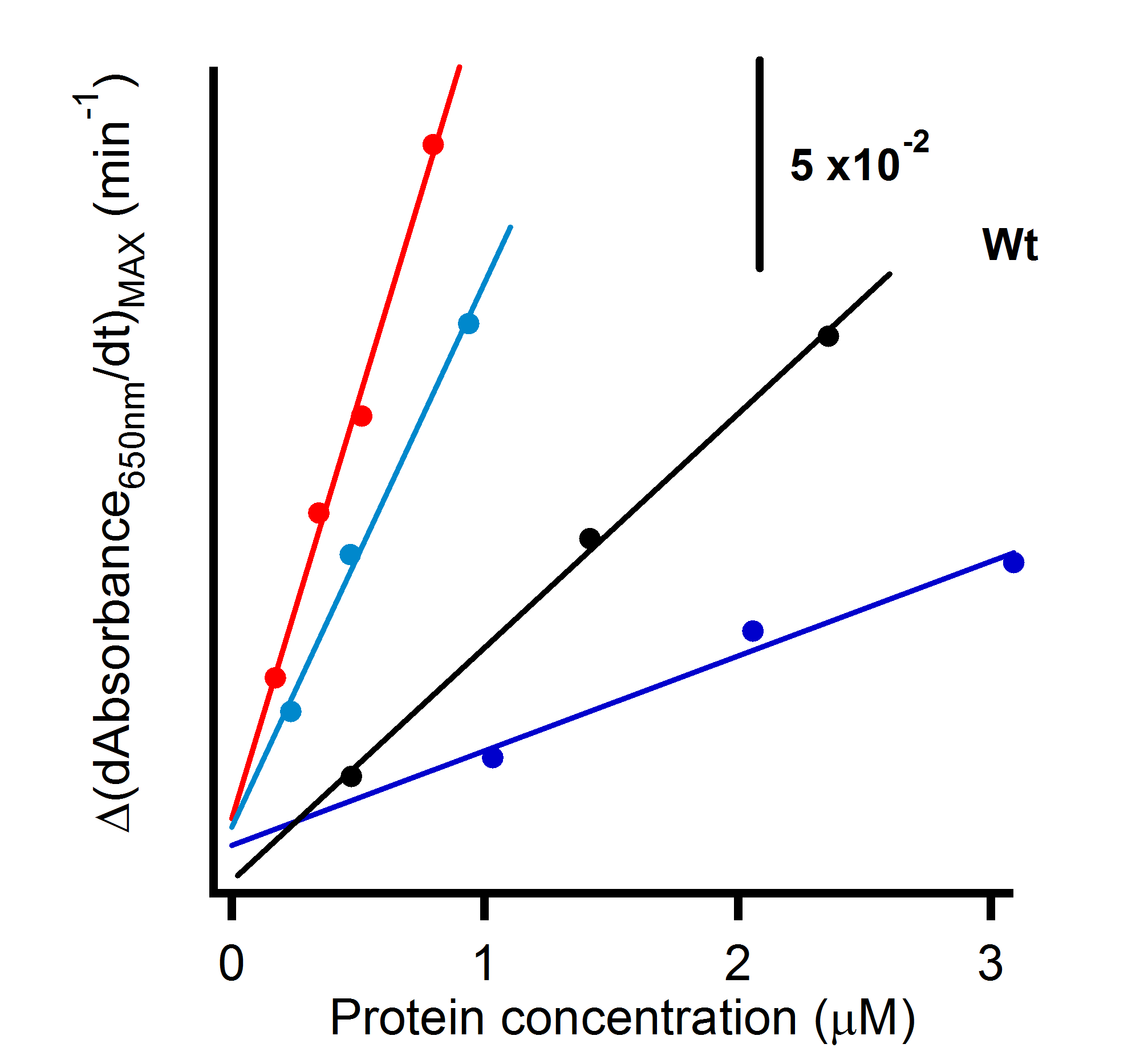

Supplement: Figure S4 — Determination of the reductase activity of thioredoxin variants. Specific activity is determined from the slopes of plots of activity (maximum value of dA650/dt) versus protein concentration. Several representative examples are shown, including wild-type thioredoxin from E.coli and several variants. (TIF) [file pcbi.1002558.s004.tif]

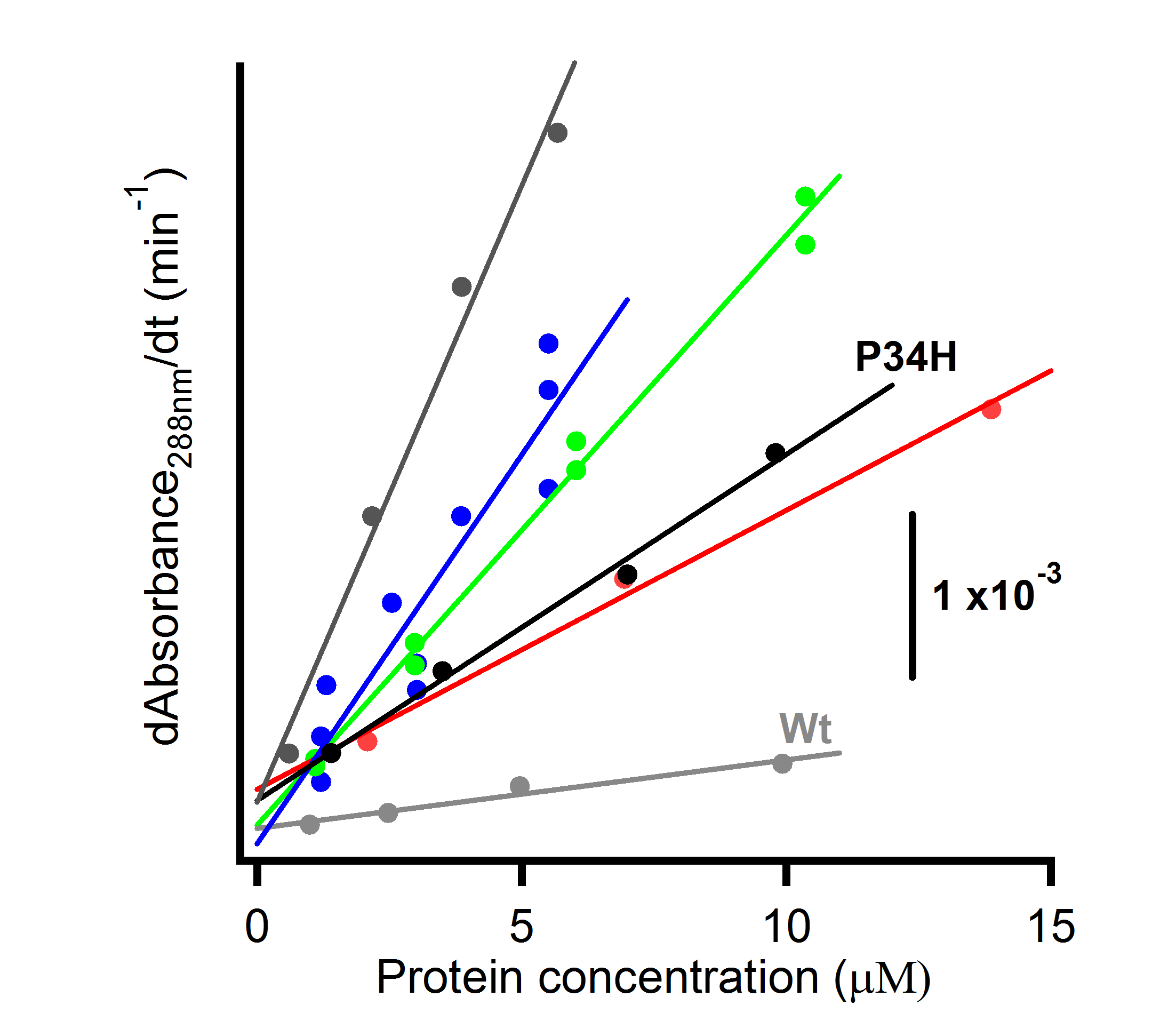

Supplement: Figure S5 — Determination of the catalysis of oxidative folding activity of thioredoxin variants. Plots of recovered RNase activity from fully reduced protein versus thioredoxin variant concentration. Specific activity is calculated as the slope of these plots. Recovered RNase activity is measured by the initial rate of the change of the absorbance at 288 nm that accompanies the hydrolysis of 2′-3′-cCMP. Several representative examples are shown, including wild-type thioredoxin from E.coli and several variants, one of which is the P34H variant used as background for library construction. (TIF) [file pcbi.1002558.s005.tif]

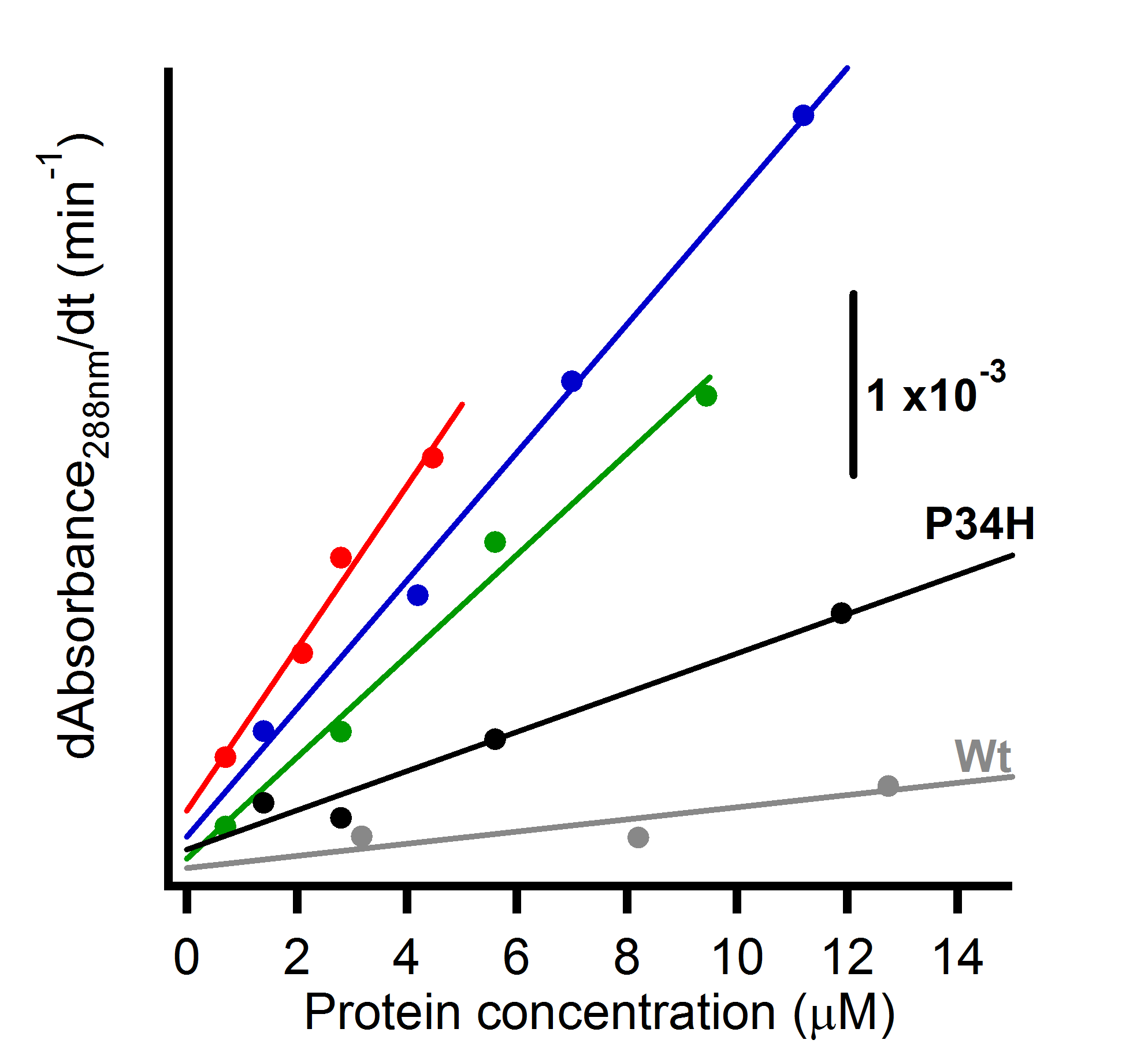

Supplement: Figure S6 — Determination of the disulfide reshuffling activity of thioredoxin variants. Plots of recovered RNase activity from disulfide-scrambled protein versus thioredoxin variant concentration. Specific activity is calculated as the slope of these plots. Recovered RNase activity is measured by the initial rate of the change of the absorbance at 288 nm that accompanies the hydrolysis of 2′-3′-cCMP. Several representative examples are shown, including wild-type thioredoxin from E.coli and several variants, one of which is the P34H variant used as background for library construction. (TIF) [file pcbi.1002558.s006.tif]

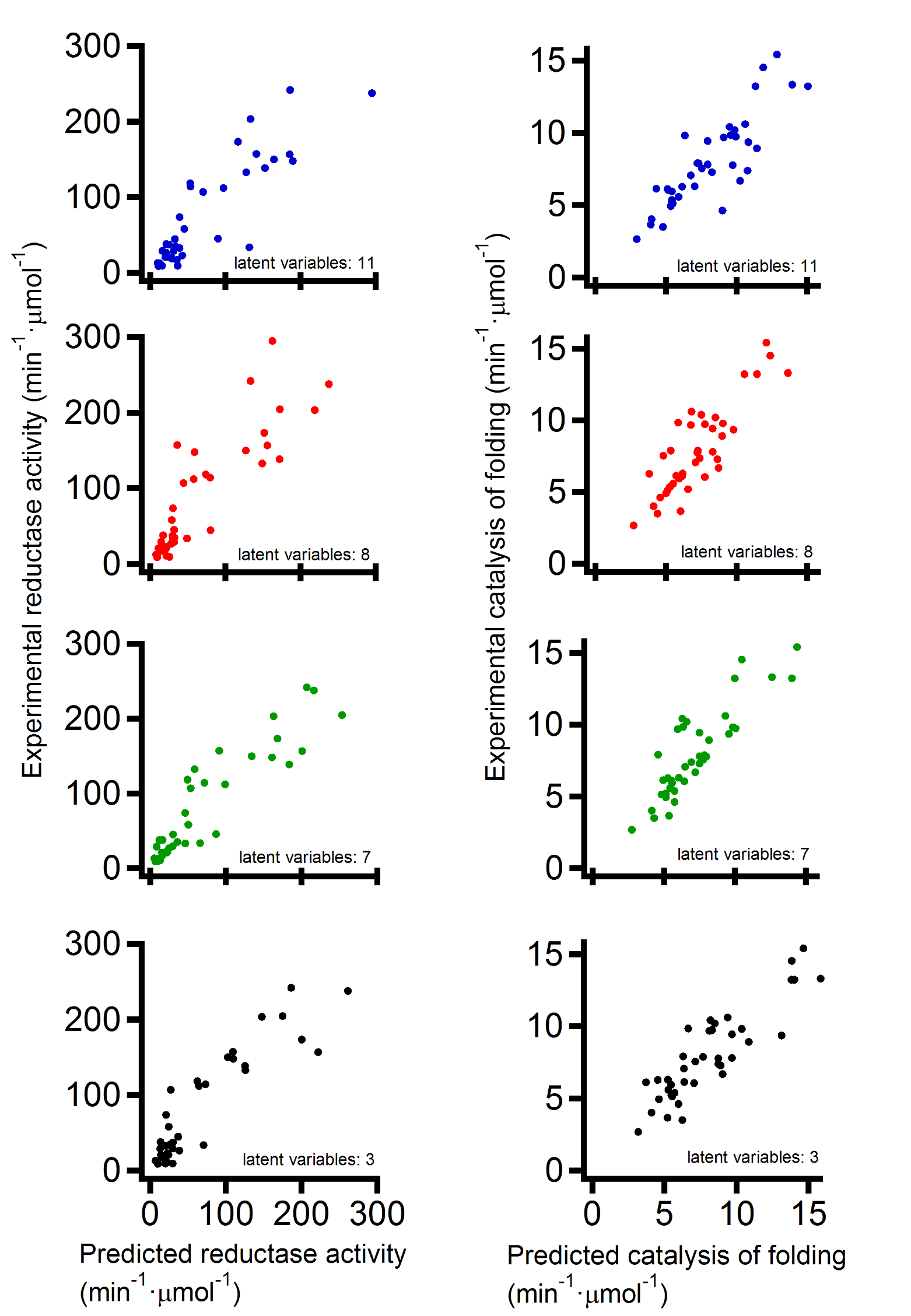

Supplement: Figure S7 — Representative examples of partial least squares fits to the 29-variants set. (Figure 5A). Actually, fits to 4 bootstrapping replicas (color-coded) extracted from that set are shown. (TIF) [file pcbi.1002558.s007.tif]
